# Approaching the quantum limit for plasmonics: linear atomic chains

**DOI:** 10.1088/2040-8978/18/7/074001

**Published:** 2016

**Authors:** Garnett W Bryant

**Affiliations:** Quantum Measurement Division and Joint Quantum Institute, National Institute of Standards and Technology, Gaithersburg, MD, 20899-8423, USA; University of Maryland, College Park, MD 20742, USA

**Keywords:** quantum plasmonics, many-body physics, excitons

## Abstract

Optical excitations in atomic-scale materials can be strongly mixed, with contributions from both single-particle transitions and collective response. This complicates the quantum description of these excitations, because there is no clear way to define their quantization. To develop a quantum theory for these optical excitations, they must first be characterized so that single-particle-like and collective excitations can be identified. Linear atomic chains, such as atom chains on surfaces, linear arrays of dopant atoms in semiconductors, or linear molecules, provide ideal testbeds for studying collective excitations in small atomic-scale systems. We use exact diagonalization to study the many-body excitations of finite (10 to 25) linear atomic chains described by a simplified model Hamiltonian. Exact diagonalization results can be very different from the density functional theory (DFT) results usually obtained. Highly correlated, multiexcitonic states, strongly dependent on the electron—electron interaction strength, dominate the exact spectral and optical response but are not present in DFT excitation spectra. The ubiquitous presence of excitonic many-body states in the spectra makes it hard to identify plasmonic excitations. A combination of criteria involving a many-body state’s transfer dipole moment, balance, transfer charge, dynamical response, and induced-charge distribution do strongly suggest which many-body states should be considered as plasmonic. This analysis can be used to reveal the few plasmonic many-body states hidden in the dense spectrum of low-energy single-particle-like states and many higher-energy excitonic-like states. These excitonic states are the predominant excitation because of the many possible ways to develop local correlations.

## 1. Introduction

Plasmons are wavelike excitations of oscillating charge density that arise in noble metals with a high density of free conduction electrons [1]. They may also occur in other systems with mobile charges such as long-chain molecules [2–4], atomic chains on surfaces [5–11] carbon nanotubes [12, 13], doped graphene [14], other complex molecules [15–18], and one-dimensional solids [19]. In extended one-dimensional systems, plasmons are charge excitations of the long-studied Luttinger liquid [20]. Plasmons can be excited by optical and infrared fields in structured materials, such as metal waveguides, gratings, nanoparticles, and atomic chains, just as radiowaves drive current in an antenna. The collective participation of a large number of charges in these plasmonic excitations gives them an intense optical response. However, rapid damping via scattering among these carriers contributes to femtosecond lifetimes and broad resonances. In the last two decades, interest in plasmonics has reemerged and exploded due to the development of nanomaterials for plasmonics, i.e., for nanoplasmonics, to confine light near surfaces, in nanoparticle gaps or near a molecule to nanoscale volumes much smaller than the diffraction limit, opening up a wide range of new applications for nanooptics [1].

Plasmons are hybrid excitations that take on the character of both the field induced around the plasmon and the oscillating charge. Classical optical physics (Maxwell’s equations and the material’s bulk dielectric function) usually provides an excellent description of the plasmonic response of nanoscale metallic systems and has been the workhorse for describing many of the nanoplasmonic effects now being exploited. While quantum effects in optics have been known for years and have spawned the field of quantum optics, it is still not obvious whether, when or how quantum effects should be important for the photonics of nanoplasmons—in particular because, unlike photons, plasmons decohere and decay in femtoseconds due to strong electron–electron scattering among the many conduction electrons participating in the plasmon, leaving little time for quantum effects to play out.

Recently, it has become apparent that quantum effects can play a significant role in plasmonics, despite rapid plasmon decay and decoherence [21, 22]. Quantum plasmonic effects can arise due to the quantum character of the participating electrons. For metal nanoparticle antennas with nanometer gaps—the antennas that provide the largest field enhancement and smallest optical mode volumes—quantum tunneling of charge across the gap distorts the surface plasmons, smearing out the local fields and limiting the useful optical enhancement [23]. In nanoparticles so small that size quantization becomes important, the broadband response of a classical plasmon breaks up into a series of discrete excitations. Some are still surface plasmons, others are single-particle-like excitations, and others have mixed character [24–26]. Similarly, in short atomic chains, quantum effects will play a critical role in the collective, plasmonic excitations.

Quantum effects should also appear because plasmons are photonic. In the last decade, a variety of quantum optics experiments have shown that plasmons can behave just like photons behave in the quantum limit with quantum coherence also surviving in the plasmonic structures [21, 22, 26]. Plasmonic nanoantennas, nanoguides, and even atomic chains, could also provide nanoscale pathways for directing optical communication between local quantum emitters. Such plasmonic guiding would extend optical quantum communication, now done with photonic structures and optical cavities, to the nano or atomic scale, with orders of magnitude better spatial resolution and with addressing that dramatically beats the diffraction limit for optics, but only if the quantum character of the information is preserved while being carried by the plasmons.

A prototypical realization for this nanoscale, optical quantum information transfer uses hybrid systems of metallic nanoparticles (MNP) linked to quantum emitters such as atoms, molecules, and quantum dots with plasmons in MNPs moving qubits from emitter to emitter. When the nanohybrid is strongly driven by a light field, the quantum coherent coupling between the light and the emitter becomes important and the dynamics of the nanohybrid can be dramatically different [27–41]. Descriptions of strongly driven nanohybrids with the emitter treated quantum mechanically and the MNP response treated classically predict a nonlinear Fano effect, bistability and induced transparency [27–34]. Recently, these nanohybrids have been described with a full quantum mechanical treatment with quantized nanoparticle plasmons [35–41]. Strong coupling and the nonlinear Fano effects also appear. Qubit entanglement, superabsorption, and cloaking are possible. However, some of the promising predictions of the quantum/classical models, such as the bistability, disappear in the fully quantum treatment [35, 41]. To date, quantized plasmons in nanohybrids have been introduced by directly quantizing the optical near fields induced around the nanoparticles by the classical plasmonic modes, just as the classical optical modes in a cavity are quantized [35–38], and broadening each quantized mode by the mode lifetime to reproduce the classical response. Quantized plasmons have also been described by introducing a collection of local oscillators with different frequencies to reproduce the broad classical response of the plasmons [39–41].

These approaches exploit the photonic character of the plasmonic excitations to implement the quantization. However, the material, electronic character of the plasmonic excitations should also be accounted for. For bulk systems with translational invariance, quantized plasmons are typically implemented, as done for lattice vibrations or other waves, by quantizing the charge density oscillations in the system [42]. A similar approach has been used for spherical nanoparticles [43]. However, these approaches face significant challenges for nanoscale systems where single-particle-like transitions can be strongly mixed with collective excitations with many participating electrons. It need not be clear which excitations should be quantized, whether they are fermions or bosons, or even which excitations are plasmonic. The effects of size quantization in small systems further distort the character of the excitations and mix the states [2, 24–26]. Resolving these issues for small systems is also complicated by the number of electrons involved. The systems are neither many-electron systems that can be treated with bulk many-body theory, nor are they simple few-electron systems. As a first step, clear characterization of the excitations must be made and the single-particle-like and collective excitations must be distinguished. We can then address the issue of how these excitations should be quantized, whether they are fermionic or bosonic, and whether excitations are independent or strongly coupled to each other. In this paper, we address this first step and show how we can distinguish the different types of optical excitations in atomic-scale systems.

In the following, we exploit exact calculations to study the excitations of linear chains of atoms. Linear atomic chains, such as atom chains on surfaces, linear arrays of dopant atoms in semiconductors, or linear molecules, provide ideal testbeds for studying collective excitations in small atomic-scale systems. To focus on the essential physics, a simple model Hamiltonian is used to describe the atomic chain. Exact calculations to find all of the excitations are possible for short chains with only a few atoms. The exact calculations provide the possibility of investigating how quantization appears without having to impose it, as is typically done. The goal here is to show how single-particle and collective excitations can be distinguished and to identify any plasmonic excitation in the spectrum of collective excitations. In section 2, we describe the model that we use for interacting electrons in short atomic chains and introduce several criteria to define plasmonic states in small atomic systems. In section 3, we describe the results for short linear chains, showing which criteria are effective at identifying plasmons. Having useful criteria is surprisingly important because only a few quantum plasmonic states appear in the full excitation spectra of linear atomic chains. We end with a discussion and conclusions in section 4.

## 2. Theoretical details

Time-dependent density functional theory (TDDFT) [44] has often been used to describe linear atomic chains [2–4, 9, 11], more complex molecules [15–17], and small metallic nanoparticles [24–26, 45, 46] to determine the time evolution and optical response of interacting electron systems. The results can be used to distinguish single-particle-like and plasmonic (i.e., collective) excitations. However, TDDFT does not tell us how these material excitations should be quantized, nor does it tell whether the excitations are fermions or bosons, or whether multiple plasmonic excitations are independent, like bosons, or coupled. Moreover, as we will show, TDDFT fails to find many of the electronic excitations of a linear atomic chain with strongly interacting electrons.

To address these quantization issues without imposing the form of the quantization as constraints the theory must obey, we need a full quantum mechanical theory of these excitations that finds all of the actual many-body eigenstates of the system rather than just the charge densities, as in DFT. With the full eigenstates, we can determine how they are quantized. To see what such a full theory could reveal, we studied some simple models for interacting electrons in small atomic-scale systems. The aim is to study systems small enough that they can be analyzed exactly. This limits the models to systems with a small number of electrons. Here we study a linear chain of atoms, with electrons hopping from atom to atom along the chain. Such a model describes atomic chains on surfaces and linear molecules. Most importantly it should provide insight on how collective excitations arise in these systems and which of the collective excitations are plasmon-like.

**Figure 1.**
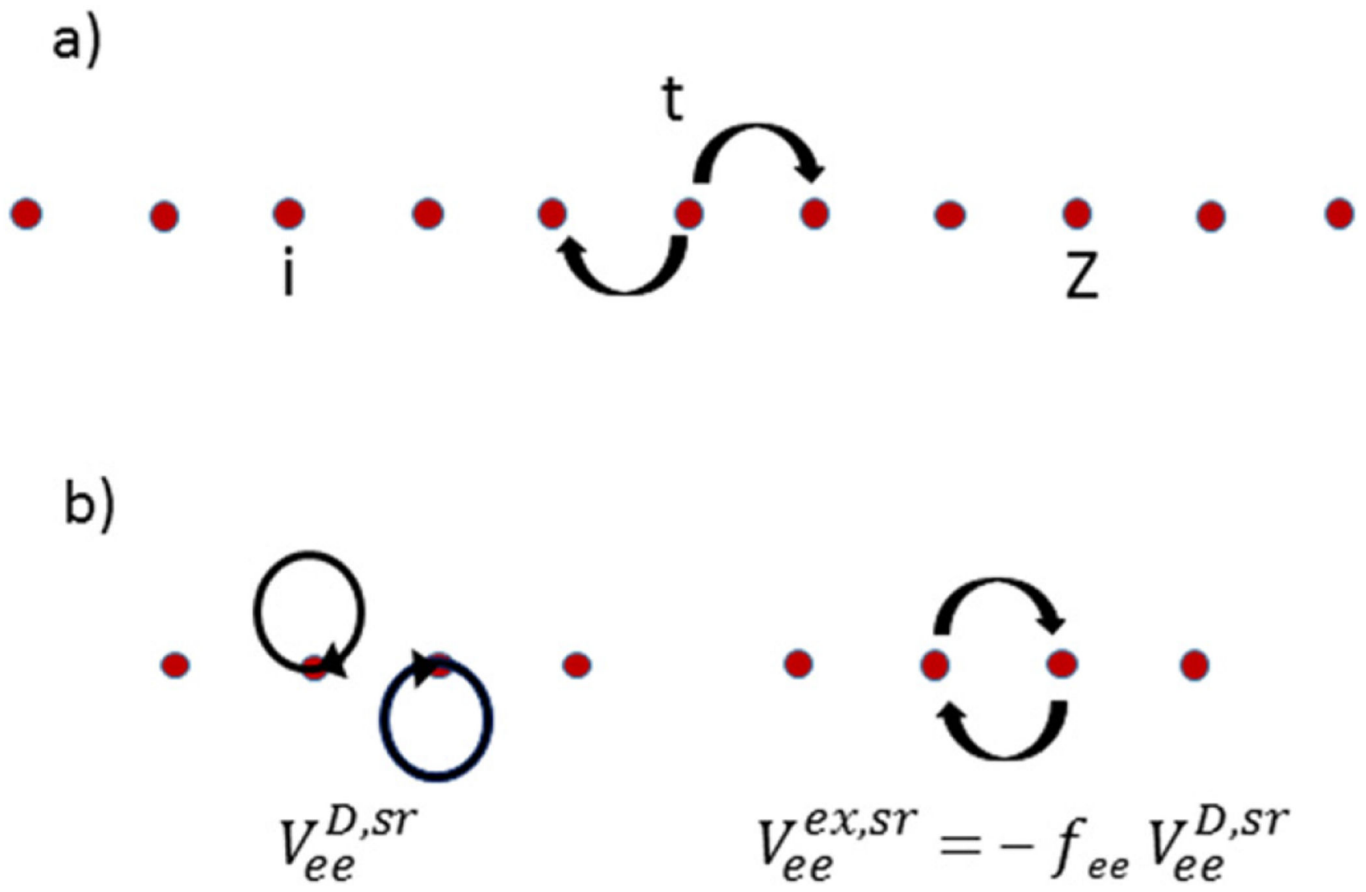
Schematic (a) of the linear atomic chain with nearest-neighbor hopping *t* between sites *i* with nuclear charge *Z*. (b) A schematic for the direct (D) short-range (sr), nearest-neighbor electron–electron interaction $V_{\mathrm{ee}}^{D,\mathrm{sr}}$, and the corresponding short-range exchange (ex) interaction $V_{\mathrm{ee}}^{\mathrm{ex},\mathrm{sr}}$. A complete circle represents coupling to the electron density at that site.

Here we consider a single-band model for the linear chain of atoms with one spinless electronic state on each atomic site and short-range, nearest-neighbor hopping *t* between atoms (figure 1(a)). If there are *N*_s_ sites in the chain and *m*_e_ spinless electrons in the system, then there are $\left(\begin{array}{c} N_{s}\\ m_{e} \end{array}\right)=\frac{N_{s}!}{m_{e}!(N_{s}-m_{e})!}$ many-body states in the system. In a charge neutral system, each site has a nuclear charge *Z* = *m*_e_/*N*_s_. An electron at site *i* interacts with the nuclear charge *Z* at site *j* via the attractive Coulomb interaction

$$V_{\text{nuc}}(i,j)=-\lambda_{\text{nuc}}Z∕(|i-j|+\xi_{\text{nuc}}),$$

where λ_nuc_ is a scale factor that includes any dielectric screening and the length scale for the site separation and *ξ*_nuc_ is a cutoff that accounts for the orbital spread of the electron orbital on a site. An electron at site *i* interacts with an electron at site *j* via the repulsive direct Coulomb interaction

$$V_{\mathrm{ee}}^{D}(i,j)=\lambda_{\mathrm{ee}}∕(|i-j|+\xi_{\mathrm{ee}}),$$

where λ_ee_ is the scale factor that includes any dielectric screening and the length scale for the site separation and *ξ*_ee_ is the cutoff that accounts for the spread of the electron orbital on a site. In this paper, no attempt is made to explicitly model a particular material. That would require atomic orbitals to properly define Coulomb integrals and hopping matrix elements. Instead, we use the simple model with λ_ee_ and λ_nuc_ as variable parameters because it allows us to investigate the full range of Coulomb interactions. The effects of electron-electron interaction are balanced by the electron nuclear interaction when λ_ee_ = λ_nuc_. The noninteracting, single-particle limit is obtained for λ_ee_ = 0. All results presented here were obtained for *ξ*_ee_ = *ξ*_nuc_ = 0.5. Results obtained for other cutoffs are similar.

In the model described above, we include the direct Coulomb interaction $V_{\mathrm{ee}}^{D}$ for interaction between the electron densities at sites *i* and *j* (see figure 1(b)). Short-range exchange, here taken to be the exchange between electrons on neighboring sites because on-site interaction is not possible for spinless electrons, can also be included. An explicit form for the atomic orbitals would be needed to directly determine this nearest-neighbor exchange. Because we do not make a choice for the atomic orbitals, we assume that the exchange is included by a reduction of the scaling $\lambda_{\mathrm{ee}}^{\mathrm{nn}}$ for the interaction between nearest-neighbor electrons $\lambda_{\mathrm{ee}}^{\mathrm{nn}}=\lambda_{\mathrm{ee}}(1-f_{\mathrm{ex}})$, where *f*_ex_ is the fraction by which the nearest-neighbor electron–electron is reduced by exchange (figure 1(b)). Interactions where one electron remains fixed and the other electron hops between neighboring sites corresponds to a density-dependent nearest-neighbor hopping. We assume that the single-particle hopping *t* dominates the hopping and that this density-dependent hopping can be ignored. All other scattering contributions from the electron–electron interaction involve even longer range hopping and are ignored. In this simple model, the two parameters, λ_ee_ and *f*_ex_, describe the strength of the electron–electron interaction.

For this model, all electronic states can be determined by standard numerical diagonalization techniques when the system has less than 10 000 states. Here we consider a half-filled band of spinless electrons (*m*_e_ = *N*_s_/2). In that case, we can consider chains with up to 16 to 20 atoms. For longer chains or when electron spin is included, it becomes impractical to determine all of the many-electron states. Instead, we must use diagonalization techniques that find limited parts of the spectrum. Here we focus on the smaller chains where all excitations can be found and analyzed. This allows us to investigate the characteristics that will distinguish collective excitations from single-particle-like excitations and to identify those collective excitations that are plasmon-like. Developing this insight will allow us to study larger systems where we must make choices about which parts of the spectrum to investigate to find the plasmon-like excitations.

Exact solutions for small systems provide a framework to address questions about the quantization. However, the first step is to see if the excitations have well-defined characteristics that can distinguish them as collective or single-particle and then to decide which collective excitations are plasmonic. As already discussed [24–26], excitations in nanoscale systems can be strongly mixed with both single-particle and collective/plasmonic characteristic. Here we follow an approach recently developed by Bernadotte *et al* [2] to identify single-particle-like and plasmonic excitations in TDDFT calculations for short linear molecules. From an analysis of the optical response function, they found that excitations of a chain of Na atoms driven by an optical field could be identified as single-particle or plasmonic. To make this identification, they varied the scale of the Coulomb interaction in the TDDFT using the same scale factor λ_ee_ we use to define the electron–electron repulsion in our model. In their TDDFT calculations, many excitations with weak response were identified as single-particle excitations. Single-particle-like excitations were characterized by excitation energies that depended only weakly on the Coulomb scale factor λ_ee_. In addition, a few excitations had strong response and excitation energies that scaled with the square root of the Coulomb interaction strength. This square root scaling is expected for plasmonic excitations. When these plasmon-like excitations cross the single-particle-like excitations, the excitations mix.

Related calculations that use a time-dependent configuration-interaction approach to study a chain of H atoms [47] also find a dominant driven mode that is plasmonic. However, in these calculations, this plasmonic mode occurs at energies below the lowest single-particle transition and should not be a transition from the many-body ground state. Moreover, this plasmonic mode only appears for high driving intensity, further suggesting that the mode is a plasmonic response of the induced charge of the state initially excited by the driving field. Although the model we use is meant to be general because no attempt is made to use parameters for a specific atomic system, the general features of our model fit most closely to the linear chain of H atoms.

We consider several criteria to analyze and characterize states. First, following Bernadotte *et al* [2], we calculate the spectra as a function of λ_ee_ to see if the states separate into different classes, some with a stronger dependence on λ_ee_, with these states passing through a denser spectrum of states weakly dependent on λ_ee_. This would give a starting point to distinguish single-particle-like excitations from collective excitations.

This analysis says nothing about oscillator strength of each excitation. For each excited many-body state *n*, we define the transition dipole moment between many-body ground state 0 and excited state *n* (assuming a driving field along the chain axis) as $D^{0n}=\langle 0|\sum_{i}n_{i}(i-i_{\text{mid}})|n\rangle$, where *i*_mid_ is the midpoint of the chain and *n_i_* is the number of electrons at site *i*. Since the position operator has odd parity about the chain midpoint, the dipole moment only connects states with opposite parity. Only half of the states will have the odd parity needed to couple to the ground state. Moreover, the position operator is a single-particle operator. It can only connect the many-body ground state to excited many-body states that have some projection onto the space of many-body states with only a single one-electron transition. In the limit where λ_ee_ → 0 and each many-body state is a determinant made from one-electron states, only a few of the lowest energy many-body states will be made with only a single one-electron transition. These are the only many-body states that couple to the many-body ground state via the dipole moment when λ_ee_ → 0. As the electron-electron interaction is turned on, the low-energy many-body states constructed from configurations that have only a single one-electron transition will be mixed into some of the higher-energy many-body states, giving these many-body states some single-particle-like characteristic that allows them to become dipole active. A strong transition dipole moment is a characteristic expected for plasmon-like optical excitations, but single-particle transitions also can have a strong transition dipole moment.

A third criterion for characterizing a many-body state *n* is its electron distribution: *ρ^n^*(*i*) = 〈*n*|*n_i_*|*n*〉. Excitations that are plasmon-like should have an induced electron distribution Δ*ρ^n^*(*i*) = *ρ^n^*(*i*) − *ρ*^0^(*i*) that piles up toward the ends of the chain. Electron distribution can be used to inspect the character of individual, selected states. It is more difficult to use this criterion to inspect all states in a spectrum.

**Figure 2.**
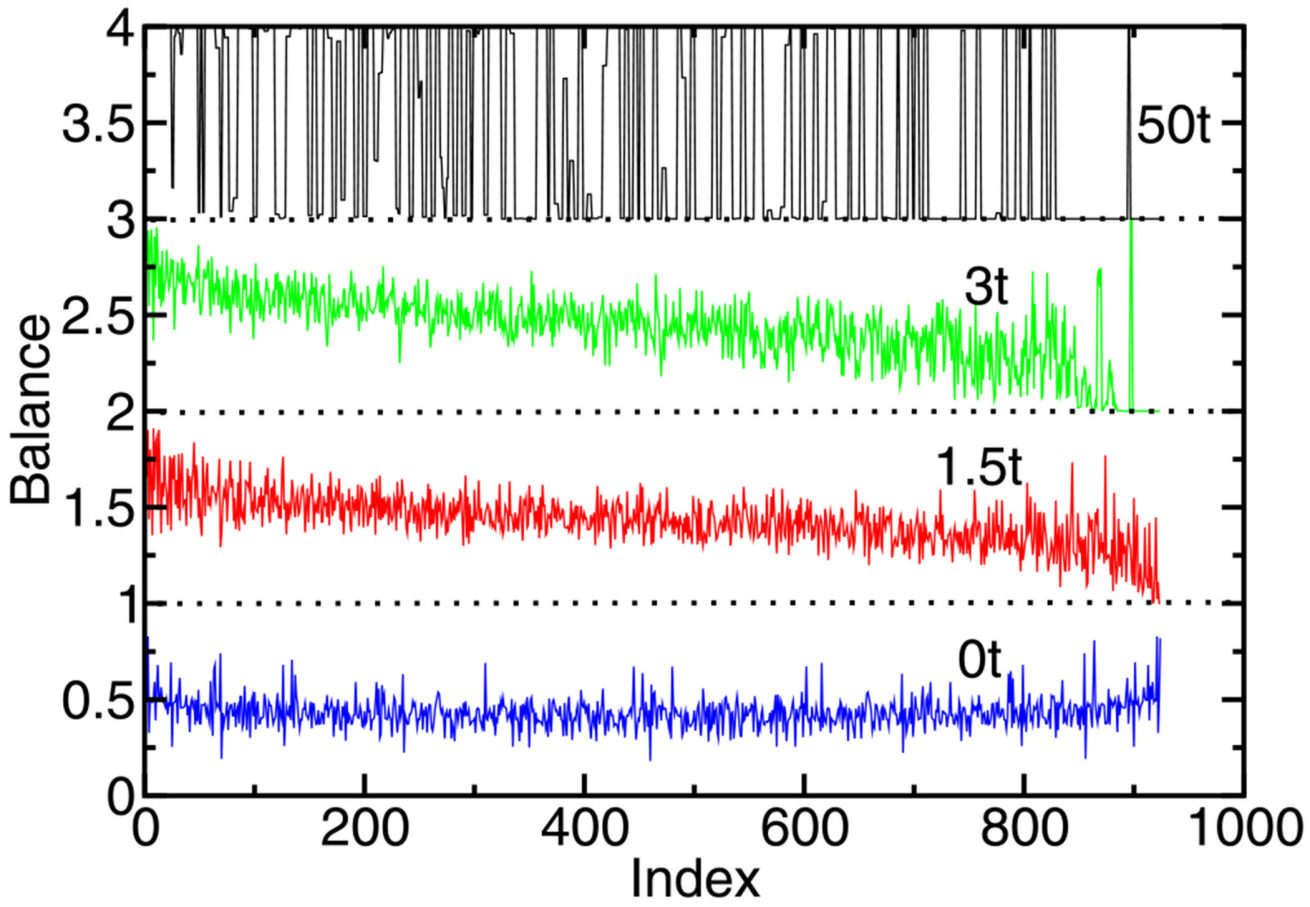
The balance of every many-body state in a 12-atom, 6-electron linear atomic chain for electron–electron interaction strength λ_ee_ = 0*t* (blue), 1.5*t* (red), 3*t* (green), and 50*t* (black) without short-range exchange. The state index ranges from 1 (lowest energy) to 924 (highest energy) for this chain. The balance varies between 0 and 1. For clarity, each curve has been offset by 1 from the other curves. Each dotted line shows the corresponding baseline. When the balance curve falls below 1, the state is only partially balanced.

More quantitative measures are needed that can easily characterize the electron distribution of every state. Here, we introduce the balance of each many-body state. The balance *B_n_* of state *n* is defined as $B_{n}=\underset{\text{states}\;b}{\sum \;\text{balanced basis}}{|\langle b|n\rangle |}^{2}$. Here the many-body state *n* is projected onto all many-body basis states *b*, which are balanced. In the site basis (the basis states for all possible arrangements of *m*_e_ electrons on *N*_s_ sites), a basis state is balanced when each half of the chain has *m*_e_/2 electrons. A many-body state is fully balanced, always with the same number of electrons on each half of the chain, when *B_n_* = 1. A state is fully unbalanced when *B_n_* = 0 and half of the chain has more electrons than the other half in every configuration that contributes to the many-body state. Intuitively, we would expect plasmon-like states to be at least partially unbalanced. Figure 2 shows the balance for each state of a 12-atom chain with 6 electrons for different values of λ_ee_. For weak interactions (λ_ee_ → 0) the balance is close to 0.5 and does not give a clear distinction between states. In this interaction regime, there is no interaction-energy gain for spreading the charge out equally on both sides of the chain and no interaction-energy cost for charge to accumulate on one side of the chain. Moreover, there is little correlation in the many-body wavefunction. As a result, there is no reason for the states to be strongly balanced or unbalanced. For λ_ee_ ≈ 1.5*t*, high-energy states (i.e., states with high index) become more unbalanced while low-energy states become more balanced. For λ_ee_ ≈ 3*t*, a clear distinction between fully balanced and fully unbalanced states develops for the highest energy states. For strong interaction (λ_ee_ ≈ 50*t*), there is also a clear distinction between balanced and unbalanced states at low energies. Most of the low-energy states are fully balanced, with only isolated clusters of a few states that are unbalanced. All many-body states close to the many-body ground state are balanced. This suggests that balance is a way to characterize the states in strongly interacting systems. In practice, this definition of balance is only useful for chains with an even number of atoms and electrons. States in chains with an even number of atoms but an odd number of electrons will always be unbalanced. For chains with an odd number of atoms, the interpretation of balance is less clear for states with charge on the central atom. While balance is used here for a one-dimensional, linear chain, it could also be applied to two- and three-dimensional symmetric systems, provided balance is defined relative to a direction with parity symmetry.

In the common picture of a plasmon in metallic nanoparticle, charge is driven back and forth across the particle in phase with a driving field. Strong optical response occurs if the frequency of the oscillating charge is resonant with the driving field. To exploit this expectation, we use hrst-order time-dependent perturbation theory to model the dynamical response of the atomic chain to a driving field with frequency *ω*. We assume that the field *F* is along the chain and coupled to the chain dipole moment. It is a straightforward exercise in hrst-order time-dependent perturbation theory to hnd the time-evolving many-body wave function Ψ(*t*) that starts at *t* = 0 in the many-body ground state. For any operator O, the time-evolving induced change in its expectation value is

〈Ψ(t)∣O∣Ψ(t)〉−〈0∣O∣0〉=2∑n⩾1〈0∣O∣n〉〈n∣V∣0〉×Im[e−iΔn0−t∕2sinΔn0+t∕2Δn0++e−iΔn0+t∕2sinΔn0−t∕2Δn0−]

where $\Delta_{n0}^{\pm }=E_{n}-E_{0}\pm \omega$ (with ℏ = 1), *E_n_* and *E*_0_ are the many-body energies and $\langle n|V|0\rangle =\langle n|F\sum_{i}n_{i}(i-i_{\text{mid}})|0\rangle$ is the matrix element for interacting with the driving field *F* coupled to the transition dipole moment, which determines the strength of the driving. When the transition energy between state *n* and the ground state is nearly resonant with the driving frequency

〈Ψ(t)∣O∣Ψ(t)〉−〈0∣O∣0〉≈〈0∣O∣n〉〈n∣V∣0〉tsinωt

the induced change grows in phase with the driving field. The strength of the induced change in *O* is determined by the transition matrix element for that operator 〈0|*O*|*n*〉 rather than the matrix element 〈*n*|*O*|*n*〉 for that operator. When *O* is the number of electrons on half of the chain, the transition matrix element tells how much charge is transferred back and forth across the chain in phase with the driving field. In the following, we refer to this as the transfer charge. The total response is proportional to the product of the transfer charge and the transition dipole moment. We refer to this as the dynamical response. This response in the time domain is directly related to the response function in the frequency domain.

In the following we use all of the criteria discussed here to characterize the states. No one criterion is sufficient to define a plasmon-like state. Both single-particle-like states and collective states can have a strong transition dipole moment or a large transfer charge. Balance provides a characterization of states in strongly interacting systems, but not when the interactions are weak. However, we will show that, at least for strongly interacting systems, there are only a few states that look plasmonic based on all of the criteria. These are the states that we will consider to be plasmonic.

## 3. Identifying plasmonic excitations in atomic chains

**Figure 3.**
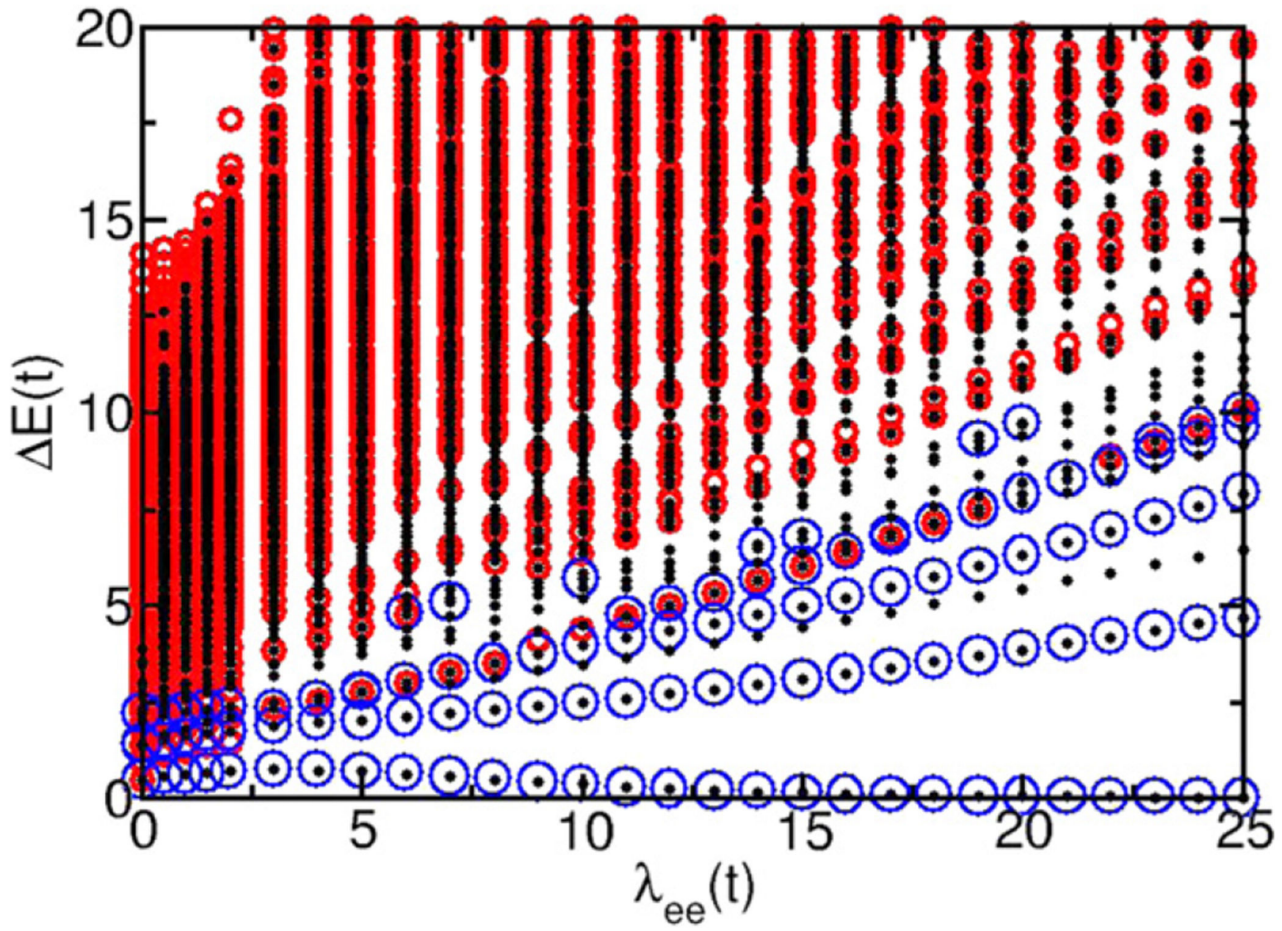
Optically active many-body excitations in the low-energy spectra of a 12-atom, 6-electron linear atomic chain as a function of the electron-electron interaction strength λ_ee_. Short-range exchange is not included. The small (black) dots indicate all excitations with finite oscillator strength. The blue, thin circles are the excitations with the largest oscillator strength. The red, thick circles indicate states with balance less than 0.5.

Here we illustrate our results by discussing chains with 12 atoms and 6 electrons. This system has a half-filled band of spinless electrons and should be metallic. Initial results were presented in [26]. To better identify the optically active single-particle-like and plasmonic/collective excitations, we eliminate from the spectra all excitations that are dipole forbidden. This eliminates all excitations that have the same parity as the ground state and eliminates all excitations made solely from configurations with multiple one-electron excitations. The excitation spectra of optically active states is shown as a function of λ_ee_ without short-range exchange (*f*_ex_ = 0) in figure 3. All excitation energies are shown relative to the many-body ground-state energy. The full spectrum, including both optically bright and dark states, 924 states in total, is more than twice as dense. The spectra for different chain lengths have a similar energy span. The main difference is the density of states. At λ_ee_ = 0, there is no electron-electron repulsion and no collective response. Each many-body excitation is made from one configuration with a single or multiple one-electron excitations (in the following discussion, one-electron excitation refers to the states of a chain with a single electron). Only many-body excitations made solely from configurations with only single one-electron excitation are optically active. These many-body states occur for excitation energies less than 4*t*. As λ_ee_ increases and the electron–electron interaction is turned on, the excitations become correlated, made from many configurations of single or multiple independent one-electron excitations and collective response is possible.

For the excitation spectra with 0 < λ_ee_ ⪅ 0.5*t* where the electron–electron repulsion is smaller than the hopping, the low-energy many-body excitations are single-particle-like, corresponding to one-electron excitations in a short chain.

The quantum confinement of a finite-chain length ensures that the one-electron states have discrete energies. At the highest energies, the discrete many-body excitations are those states formed by removing a single one-electron excitation from the fully excited state. In the low interaction regime for 0 < λ_ee_ ≲ 0.5*t*, these single-particle-like excitations lose oscillator strength as λ_ee_ increases while higher energy excitations above the single-particle-like transitions become optically active. The excitation energies increase weakly as the electron–electron repulsion is added. The excitation energies for a few of the states increase faster than others. However, there is no clear separation of the many-body states, as predicted by TDDFT, into a set of single-particle-like excitations that are nearly independent of λ_ee_ and another set of many-body excitations with excitation energies that scale as the square root of λ_ee_. In this range of λ_ee_, interaction is too weak to have much effect.

This weak dependence on λ_ee_ may seem surprising because the electron–electron interaction is comparable to the hopping for λ_ee_ ≈ *t*. However, the full Coulomb interaction includes both the electron–electron repulsion and the electron–nucleus attraction. The chains are chosen to be charge neutral, so the repulsive and attractive interactions compensate for each other and the net Coulomb interaction is weak for λ_ee_ ≈ *t*. The spectra for λ_ee_ up to 25*t* are shown in figure 3. For low λ_ee_, the many-body ground state electron density is uniform along the chain, as would be expected for a metallic state. For large λ_ee_ (λ_ee_ ≈ 25*t*), the first many-body excited state and the ground state become degenerate. The ground state electron density remains uniform. However, for these large λ_ee_, a small electric field along the chain axis breaks the double degeneracy of the ground state and polarizes the uniform ground state charge into a Wigner crystal where the electrons separate and localize onto every other site due to their mutual repulsion. This suggests that metallic behavior extends up to λ_ee_ ≈ 25*t* where Wigner crystallization appears. In addition, the two highest many-body excitations become degenerate for λ_ee_ > 1.5*t* with all of the charge polarized to half of the chain if a small electric field is applied. These two highest-energy states are the highest energy plasmon-like states in the spectrum. However, they are not the low-energy plasmon-like states we are trying to identify. For smaller λ_ee_, even the highest excitation has uniform electron density along the chain. These results suggest that strong collective response begins to appear for λ_ee_ > 1.5*t* but metallic behavior persists until λ_ee_ ≈ 25*t*. In this range of λ_ee_, the low-energy excitations remain well separated from the quasicontinuum and show clear linear dependence on λ_ee_. Analysis of these states shows that they are formed from many single and multiple independent one-electron configurations, indicating strong collective character.

The state balance *B_n_* can be used to further distinguish the states. Figure 3 shows which optically active states have large oscillator strength and which have large unbalance (*B_n_* < 0.5). The lowest energy unbalanced state has large oscillator strength, indicating that this is a reasonable candidate for the lowest-energy plasmonic state.

**Figure 4.**
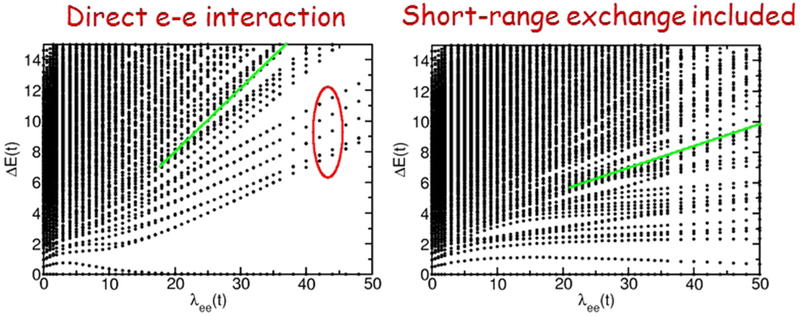
Comparison of the spectra, as a function of λ_ee_, without (left) and with (right) short-range exchange (*f*_ex_ = 0.2). The thick, green lines in each plot show the lowest-energy strongly unbalanced many-body state (i.e., balance less than 0.5). The ellipse highlights many-body states that evolve from the single-particle excitations as the interaction increases.

As shown in figure 4, when only direct electron–electron repulsion is included, the spectra split into at least two classes of many-body states, each characterized by excitation energies that increase linearly with λ_ee_ for large interaction. Even the lowest-energy many-body states, which evolve from the single-particle-like excitations, depend linearly on λ_ee_. The higher-energy many-body states split off from the low-energy states indicated by the ellipse in figure 4 and the excitation energies for the higher energy class of many-body states increase more rapidly with λ_ee_. However, there is no evidence of a class of single-particle-like excitations with energies that depend weakly on λ_ee_, and there is little mixing or crossing of the two different classes of states. This difference between the exact model and the TDDFT results remains even when balance and oscillator strength are used to filter out states and simplify the spectra. However, when short-range exchange is included, significant changes in the spectra occur, as shown in figure 4. The short-range exchange reduces the nearest-neighbor repulsion and weakens the interaction effects. As a consequence, the lowest-energy many-body states are only weakly dependent on λ_ee_. In addition, the energies of higher states also show a weaker increase with increasing interaction. This allows more higher-energy many-body states to overlap, cross, and mix with the low-energy many-body states that are weakly dependent on λ_ee_. When short-range exchange is included, the spectra for the exact model are more similar to the TDDFT predictions.

**Figure 5.**
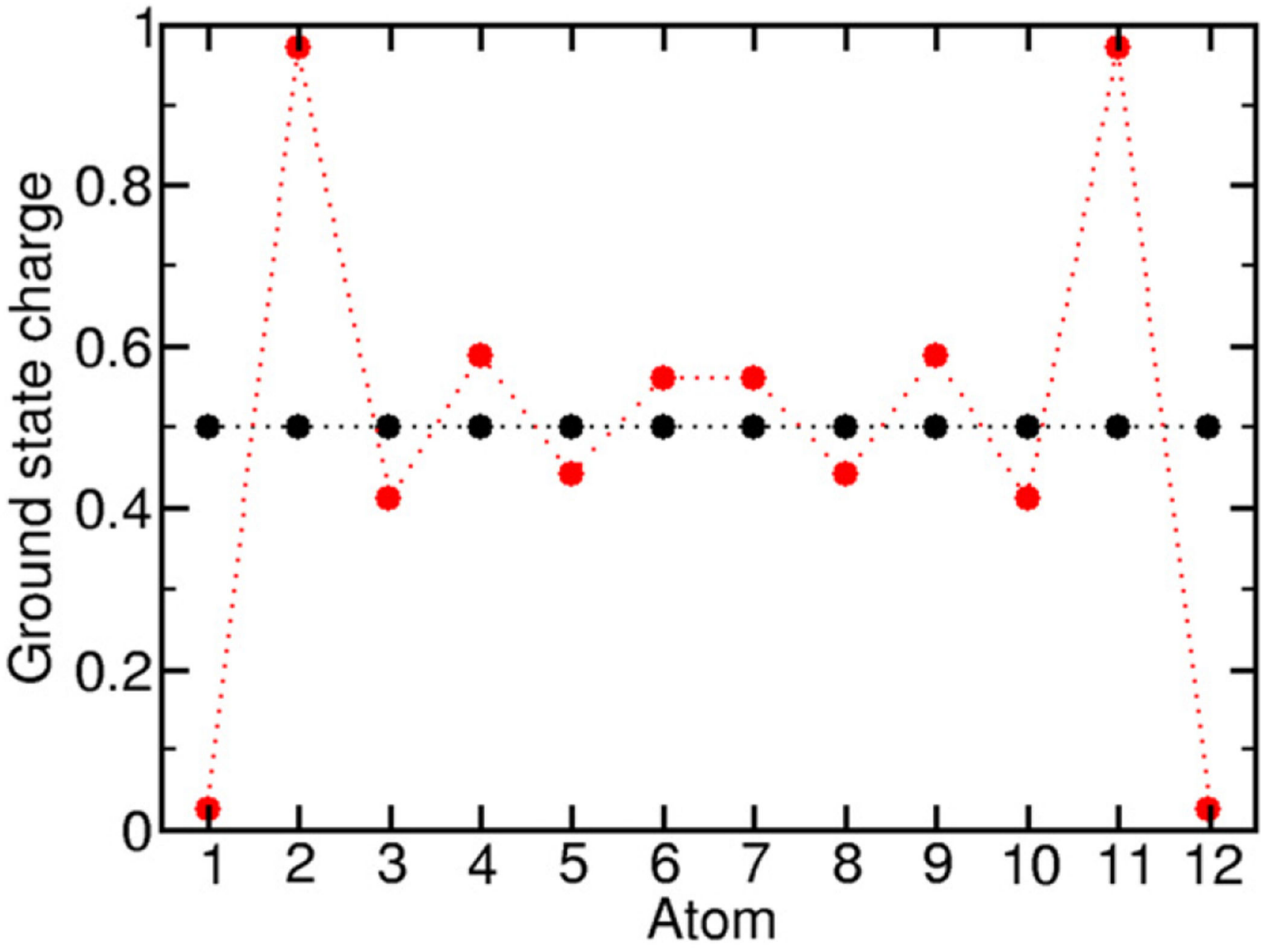
Ground-state electron density for a 12-atom, 6-electron chain with (red) and without (black) short-range exchange, λ_ee_ = 50*t*.

Short-range exchange is also important for defining the ground-state-charge density. Without exchange, the ground-state-charge density is uniform across the chain, as shown in figure 5. When exchange is included, charge at the end of the chain is pulled into the interior of the chain. This allows electrons near the end of the chain to gain from exchange coupling to nearest-neighbor electrons on both sides, rather than on just one side.

These results show that short-range exchange plays a key role in defining the many-body states. For the range of exchange (0 ⩽ *f*_ex_ ⩽ 0.2) shown in figure 4, the many-body states evolve continuously as the exchange is changed. However, just above *f*_ex_ = 0.2 a transition occurs and the many-body ground state remains nondegenerate for all λ_ee_. These effects of exchange are beyond the scope of this paper and will be discussed elsewhere.

**Figure 6.**
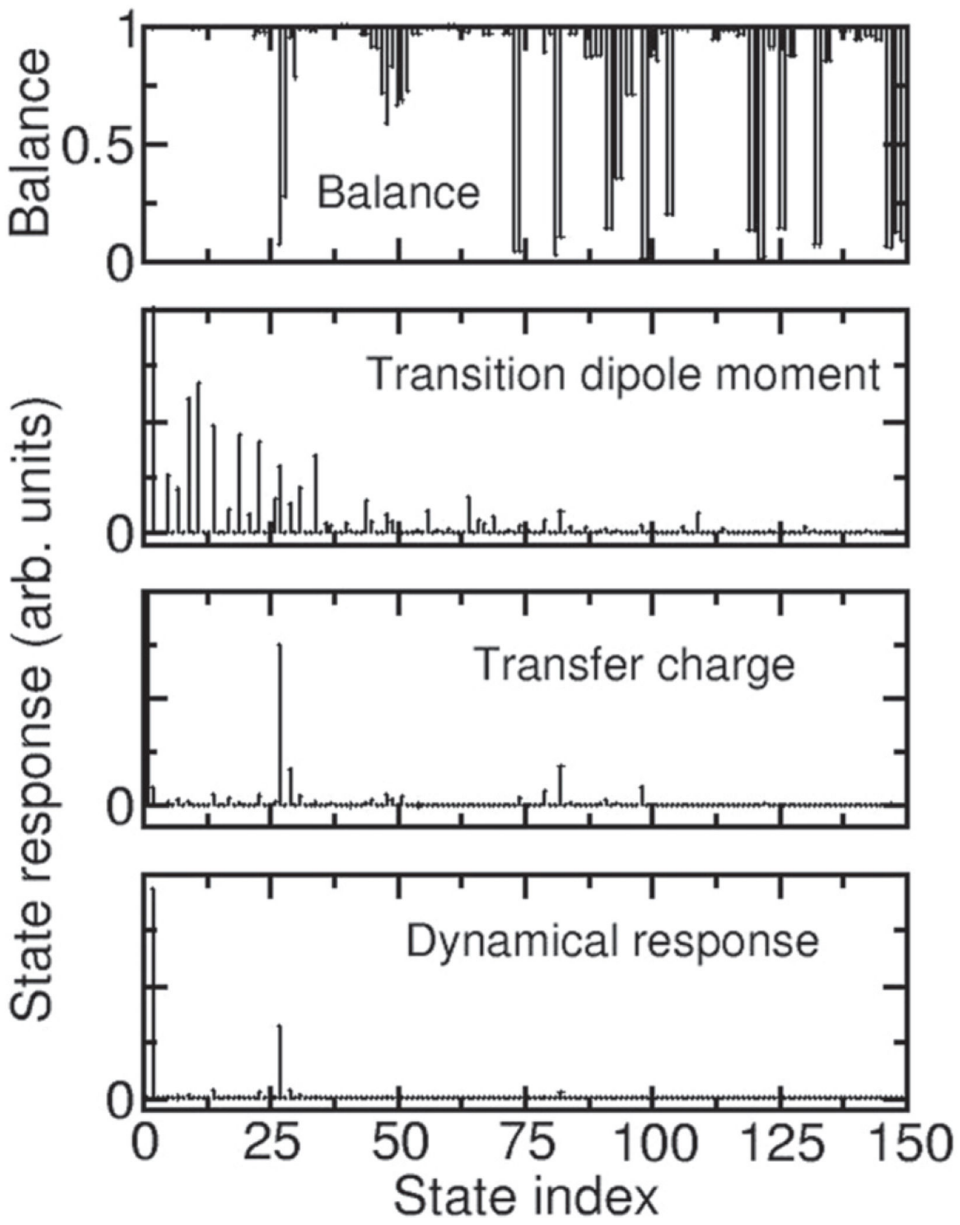
Comparison of the many-body state balance, transition dipole moment, transfer charge, and dynamical response (the product of the transition dipole moment and the transfer charge) for a 12-atom, 6-electron chain with λ_ee_ = 50*t* and *f*_ex_ = 0.2. When the balance curve falls below 1, the state is only partially balanced.

**Figure 7.**
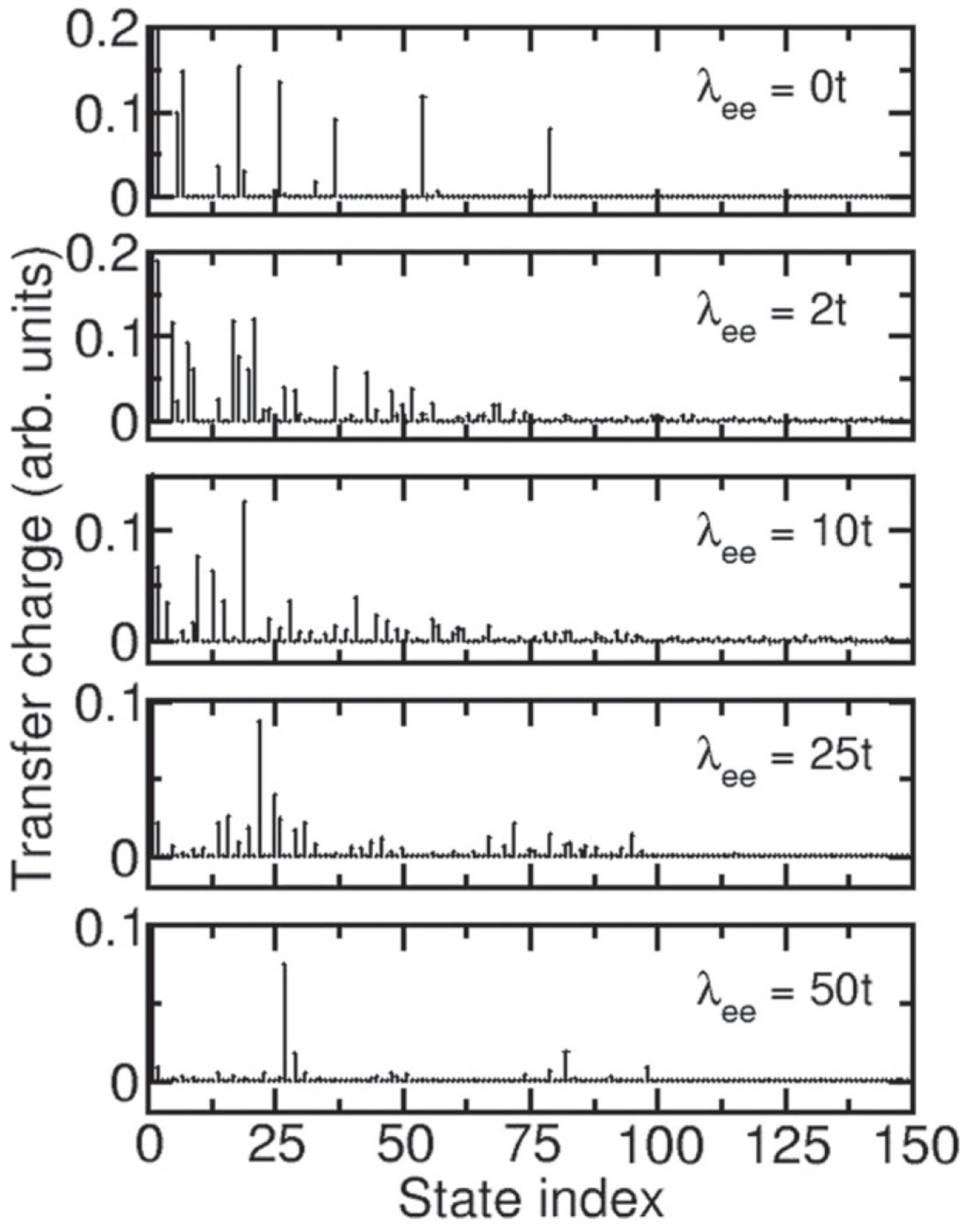
Comparison of the many-body state transfer charge for a 12-atom, 6-electron chain with λ_ee_ = 0*t*, 2*t*, 10*t*, 25*t*, and 50*t* and *f*_ex_ = 0.2.

To fully characterize these two classes of states for the case where exchange is included, we compare in figure 6 the balance, transition dipole moment, transfer charge, and dynamical response of the many-body states when λ_ee_ = 50*t*. As shown by figure 2, balance provides a useful characterization of the states for this interaction strength. The lowest-energy unbalanced many-body state also has a significant dipole moment, transfer charge, and dynamical response. In fact, only a few states have a significant transfer charge and dynamical response. This suggests that this lowest unbalanced state is the state that should be considered as the lowest energy plasmonic many-body state. The few higher-energy states that also have a significant transfer charge and dynamical response could be considered excited plasmonic states. As shown in figure 7, this identification remains possible for λ_ee_ > 10*t*. For smaller interaction strengths, many states have a significant transfer charge. This suggests that plasmonic states can be identified for λ_ee_ > 10*t*, but that it becomes problematic to make this identification for smaller interaction strengths. The solid green lines imposed on the spectra shown in figure 4 show where the lowest unbalanced state with large transfer charge occurs in the spectra.

The optical absorption of the chain is proportional to the square of the transition dipole moment. As can be seen from the dipole moments shown in figure 6, there is a broad spectrum of strong absorption peaks at low energy and much weaker absorption at higher energy. The plasmon peak contributes to this broad low-energy spectrum but single-particle and excitonic transitions make a dominant contribution. In contrast, for TDDFT [20] the plasmon peak is predicted to be the dominant contribution to the low-energy response.

**Figure 8.**
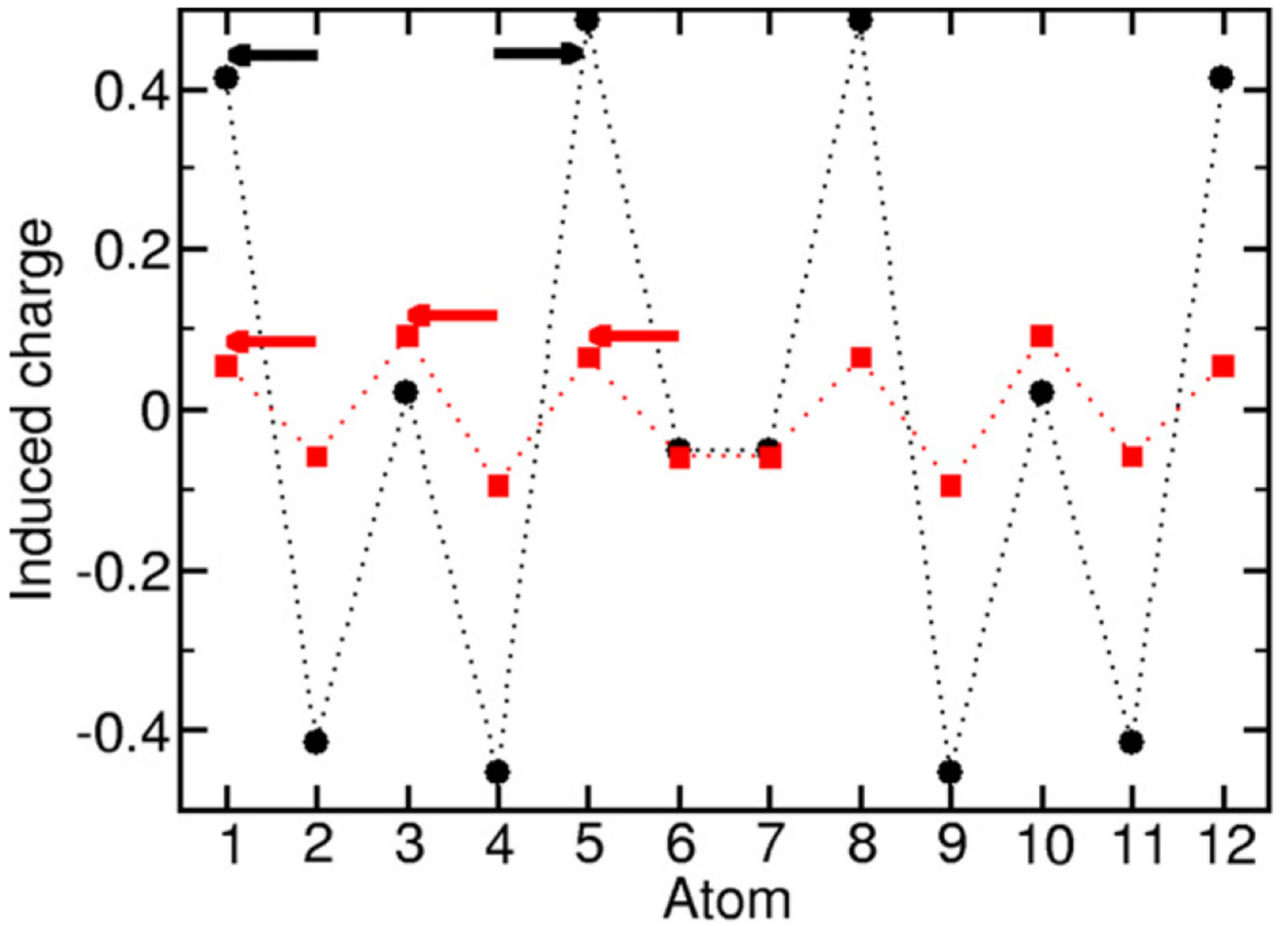
Comparison of the induced-electron density for the lowest unbalanced many-body state (red) and a typical exciton-like many-body excitation (black) in a 12-atom, 6-electron chain with λ_ee_ = 50*t* and *f*_ex_ = 0.2. The arrows indicate how the induced charge shifts.

As a final characterization of this lowest unbalanced state as a plasmonic state, we show in figure 8 the induced charge density (red curve) of the state. The corresponding arrows indicate how the induced charge shifts from the ground state. This induced charge shows the plasmonic feature of being pushed globally toward the outside of the chain and piling up toward the ends despite the exchange-induced suppression of charge at the end of the chain. For comparison, we show the induced charge density (black curve) of one of the much more numerous many-body states with negligible transfer charge. Here the induced charge shift is local. It could be considered as a multiexcitonic state with several local electron-hole pairs created. In this example, the two excitons on each side of the chain move as a breathing mode. Inspection of the induced-charge density of each of the many-body states shows that this local, excitonic, induced-charge distribution is the typical configuration. Plasmonic states are much rarer. In part, this explains why spectra for the exact models can appear very different from the predictions of TDDFT. TDDFT does not find most of these excitonic states because the local correlations needed to describe them are not fully included. The related configuration interaction calculations, which do include the lowest-order correlations in the many-body wavefunction, do exhibit a higher density of nonplasmonic states. However, the plasmons found in the configuration interaction calculations are excited state plasmons different from the plasmons found in the exact calculations.

## 4. Conclusions

To develop a quantum theory for optical excitations in atomic-scale systems, the excitations must first be characterized so that single-particle-like and collective excitations can be identified. Linear atomic chains, such as atom chains on surfaces, linear arrays of dopant atoms in semiconductors, or linear molecules, provide ideal testbeds for studying collective excitations in small atomic-scale systems. We use exact diagonalization to study the many-body excitations of finite linear atomic chains. We find that plasmonic many-body states can be identified. However, there are only a few plasmonic states, hidden in a dense spectrum with low-energy single-particle-like states and many higher-energy excitonic-like states. These excitonic states are the predominant excitation because of the many possible ways to develop local correlations. The plasmonic states are most apparent for strong interaction. For weak interaction it becomes more difficult to distinguish specific states as being plasmonic. No one criterion gives a compelling identification of the plasmonic states. However, a combination of criteria involving the transfer dipole moment, the state balance, the transfer charge, the dynamical response, and the induced charge distribution do strongly suggest which states should be considered as plasmonic. This identification of the plasmonic excitations is the first step in an effort to understand quantum plasmonics in these systems.

Thus far, the work reported here has been restricted to one-dimensional chains, spinless electrons, and chains with less than 25 atoms. These choices have been made to make exact calculations tractable. However, limitations are imposed by these choices. The full effect of exchange and Pauli blockade is lost when there is only one spin component. For systems restricted to one dimension, Pauli blockade also limits electron motion and correlation. Similarly, the kinetic energy cost is significantly higher when motion is restricted to one dimension. It will be a challenge to relax these restrictions and extend the calculations to include both spin components, allow motion and correlation in more dimensions, and consider longer systems. However, such extensions need to be pursued to understand how, for example, atomic-scale plasmons transform into nanoscale, metallic nanoparticle plasmons as system size and dimension increases or how plasmons in a finite one-dimensional chain transform into the excitations of a Luttinger liquid in an extended system.
